# Pimonidazole binding in C6 rat brain glioma: relation with lipid droplet detection

**DOI:** 10.1038/sj.bjc.6600837

**Published:** 2003-04-29

**Authors:** S Zoula, P F J W Rijken, J P W Peters, R Farion, B P J Van der Sanden, A J Van der Kogel, M Décorps, C Rémy

**Affiliations:** 1 Laboratoire Mixte INSERM U438 ‘RMN Bioclinique’, Université Joseph Fourier, Laboratoire Correspondent de CEA, Centre Hospitalier Universitaire Pavillon B, BP 217, 38043 Grenoble Cedex 09, France; 2 Department of Radiotherapy, University of Nijmegen, Geert Grooteplein 32, 6500 Nijmegen, The Netherlands

**Keywords:** glioma, hypoxia, lipid droplets, perfusion, image analysis

## Abstract

In C6 rat brain glioma, we have investigated the relation between hypoxia and the presence of lipid droplets in the cytoplasm of viable cells adjacent to necrosis. For this purpose, rats were stereotaxically implanted with C6 cells. Experiments were carried out by the end of the tumour development. A multifluorescence staining protocol combined with digital image analysis was used to quantitatively study the spatial distribution of hypoxic cells (pimonidazole), blood perfusion (Hoechst 33342), total vascular bed (collagen type IV) and lipid droplets (Red Oil) in single frozen sections. All tumours (*n*=6) showed necrosis, pimonidazole binding and lipid droplets. Pimonidazole binding occurred at a mean distance of 114 *μ*m from perfused vessels mainly around necrosis. Lipid droplets were principally located in the necrotic tissue. Some smaller droplets were also observed in part of the pimonidazole-binding cells surrounding necrosis. Hence, lipid droplets appeared only in hypoxic cells adjacent to necrosis, at an approximate distance of 181 *μ*m from perfused vessels. In conclusion, our results show that severe hypoxic cells accumulated small lipid droplets. However, a 100% colocalisation of hypoxia and lipid droplets does not exist. Thus, lipid droplets cannot be considered as a surrogate marker of hypoxia, but rather of severe, prenecrotic hypoxia.

The presence of mobile lipids has been detected by proton magnetic resonance spectroscopy (^1^H MRS) in animal and human tumours: in cultured cells (Mountford and Wright, 1988; Le Moyec *et al*, 1992), in biopsies (Lean *et al*, 1993; Kuesel *et al*, 1994a), and *in vivo* (Ott *et al*, 1993; Negendank *et al*, 1996). The location of these lipids has been widely discussed during the past years. The lipid signal was first ascribed to neutral lipids, mainly triglycerides, arranged in microdomains (25–28 nm in diameter) embedded within the phospholipid bilayer (Mountford and Wright, 1988). In 1993, Callies *et al* (1993) demonstrated a correlation between the appearance of mobile lipids in proton magnetic resonance (MR) spectra of myeloma cells, and the formation of cytoplasmic droplets (1 *μ*m in diameter). A relation between the amount of mobile lipids and the percentage of necrosis was also evidenced in proton spectra of human brain tumour biopsies (Kuesel *et al*, 1994b). All these results encouraged Rémy *et al* (1997) to make a new estimate of the size of the compartment in which mobile lipids were confined. Using *in vivo* diffusion measurements, they hypothesised a link between mobile lipids detected by ^1^H MRS, and the presence of necrosis and lipid droplets in a model of C6 rat glioma. In addition, transmission electron microscopy of tumour fragments showed increased numbers of lipid droplets with increasing sizes from the periphery of the necrosis towards the centre of the tumour (Rémy *et al*, 1997). These results were confirmed and extended by Lahrech *et al* (2001). By measuring the mean displacement of mobile lipids in C6 rat brain glioma at different diffusion times, a characteristic diameter of 4.7 *μ*m was determined for the compartment that contains these lipids (Lahrech *et al*, 2001). Consistent with this, we recently confirmed that ^1^H MRS-visible mobile lipids in C6 rat glioma arise from lipid droplets mainly located in the necrosis (Zoula *et al*, 2001). Consequently, the detection of a high intensity of ^1^H MRS lipid signal could be a signature of a very necrotic tissue and indicate the tumour grade.

In several studies, a lower amount of smaller lipid droplets has also been observed in the cytoplasm of viable tumour cells surrounding the necrosis (Freitas *et al*, 1990; Rémy *et al*, 1997; Lahrech *et al*, 2001; Zoula *et al*, 2001). Lipid droplets and/or MRS mobile lipids signal have been detected in various hypoxic and/or ischaemic situations such as acute ischaemia in cat heart (electronic microscopy) (Jodalen *et al*, 1985), human cerebral infarction (^1^H MRS) (Saunders *et al*, 1995; Hwang *et al*, 1996), tumour hypoxic zones (fluorescence microscopy and electronic microscopy) (Freitas, 1990; Freitas *et al*, 1996). It has been proposed (Freitas *et al*, 1990, 1996; Rémy *et al*, 1997) that these lipid droplets could represent a temporary storage compartment (final storage compartment if the tissue does not recover) of fatty acids in the form of triglycerides. Under hypoxic or ischaemic conditions, the fatty acids would not be degraded any more by *β*-oxidation. The harmful effects of the increase in the cell concentration of free fatty acids would be avoided by sequestering them in the form of triglycerides in lipid droplets. Thus, the accumulation of lipid droplets in viable tumoral cells located at the periphery of necrosis would result from a stress induced by the hypoxic state of these cells (Freitas *et al*, 1990, 1996; Kuesel *et al*, 1994b). This lipid accumulation would accelerate with the necrotic process (Kuesel *et al*, 1994b).

Tumour hypoxia has long been considered as a limiting factor in association with poorer outcome after radiotherapy, chemo-therapy and surgery (Durand and Raleigh, 1998; Raleigh *et al*, 1999). Almost all macroscopic transplanted tumours in rodents contain viable hypoxic cells, which are a major cause of radioresistance (Rockwell and Moulder, 1990; Morioka *et al*, 1992). Since past years, many studies have shown a sustained interest in development of techniques for detecting hypoxia. However, methods for identifying hypoxic viable tumour cells in cancer patients remain limited (Durand and Raleigh, 1998). Thus, the incidence of hypoxia in human brain tumours before and during radiotherapy is poorly understood (Parliament *et al*, 1997). The existence of a technique, which would allow a simple detection of hypoxic cells, would constitute an undeniable advantage for the diagnosis, the prognosis, the orientation and therapeutic follow-up of cancers. In this study, we hypothesised that the presence of lipid droplets is related to the occurrence of hypoxia near necrosis. To validate this hypothesis, hypoxia marker binding was analysed in relation to necrosis and lipid droplets in histological sections of C6 rat gliomas. The use of lipid droplets as an indicator of necrosis and/or hypoxia was thus evaluated.

## MATERIALS AND METHODS

### Experimental model of intracerebral glioma

Intracerebral tumours were induced by C6 cells, a cell line derived from a chemically induced malignant rat glioma (Benda *et al*, 1971). A measure of 5 *μ*l of cell suspension, 10^5^ cells, (prepared in Dulbecco's modified Eagle's medium (DMEM) supplemented with 2% glutamine and 1% antibiotics) were stereotactically injected 3 mm below the dura in the right caudate nucleus of six female Wistar rats (160–180 g) (Workman *et al*, 1998).

All operative procedures and animal care strictly conformed to the French Government guidelines (decree no. 87-848 of 19 October 1987, licenses 006722 and A38071).

### Immunohistochemistry and section analysis

A multiple fluorescence staining technique combined with a digital image analysis was used to study quantitatively tumour vascularity, perfusion, hypoxia and lipids in the same section (Rijken *et al*, 2000). Pimonidazole (1-[(2-hydroxy-3-piperidinyl) propyl]-2-nitroimidazole hydrochloride) was used as a hypoxia marker (Kennedy *et al*, 1997; Durand and Raleigh, 1998; Varia *et al*, 1998; Raleigh *et al*, 1999), Hoechst 33342 was utilised for the detection of perfused vasculature, and anticollagen type IV was used as an anatomical marker of all vessels.

Experiments were carried out by the end of the tumour development (22–25 days after tumour cell implantation). Rats were injected with a 0.25 ml solution of pimonidazole in phosphate-buffered saline (PBS, pH 7.4) (80 mg kg^−1^ of body weight; Natural Pharmaceuticals International Inc., Research Triangle Park, NC, USA) and a 0.25 ml solution of Hoechst 33342 in PBS (24 mg kg^−1^ of body weight; Sigma-Aldrich, Saint-Quentin-Fallavier, France). These markers were administrated intravenously via one of the tail veins, 60 and 1 min before killing, respectively.

Brains were quickly removed (less than 3 min after the rat death), frozen in liquid nitrogen then stored at −80°C to prevent the Hoechst from diffusing further into the tissue.

For each rat, two adjacent transversal sections (5 *μ*m thick) were cut at mid-tumour (where the tumour was the largest). To analyse the morphology of the tumour, the first section was stained with haematoxylin/eosin (HE). The second section was analysed on the digital image analysis system. This section was first scanned to obtain a composite image with perfused areas (Hoechst binding), followed by fixation in 10% buffered formaldehyde. Next, lipid droplet staining was performed with a specific lipid dye, Red Oil (Fowler and Greenspan, 1985) (Fluka Chemie AG, Buchs, Switzerland). Then, the section was incubated for 90 min at 4°C with primary antibodies: 1 : 100 dilution of a goat polyclonal antibody against collagen type IV (Southern Biotechnology Associates, Birmingham, AL, USA), and 1 : 200 dilution of a rabbit polyclonal antibody against pimonidazole both in PBS with 0.5% BSA-c (BSA-c; Aurion, Wageningen, The Netherlands). This step was followed by incubation with secondary fluorescent antibodies for 2 h: 1 : 100 dilution of the Alexa Fluor® 546 IgG (H+L) donkey anti-goat (Molecular Probes, Eugene, OR, USA) and 1 : 100 of the Fluorescein FITC-conjugated IgG (H+L) donkey anti-rabbit (Jackson ImmunoResearch Laboratories, West Grove, PA, USA) both in PBS with 0.5% BSA-c.

The sections stained with HE were evaluated by light microscopy using a Nikon Eclipse E600 microscope equipped with an Olympus DP 50 video camera and an image analysis system (analySIS) developed by Olympus (Soft Imaging System, Münster, Germany). Tumour and necrosis areas were measured and the data were classed according to the necrotic fraction, *R*, defined as the necrotic area divided by the total tumour area. Tumour perfusion, vascularity and oxygenation were analysed using the system described by Rijken *et al* (1995, 2000). Briefly, the fluorescence signals were recorded by a high-resolution intensified solid-state black and white video camera. The video signal was digitised to images with 256 grey levels, and the digital imaging application TCL-Image (TNO, Delft, the Netherlands) was used for acquiring and further processing the images. Before scanning a series of particular fluorophore, the automated scanning stage was initialised and calibrated and a threshold grey value to segment the objects from the background was determined and recorded.

The perfused areas (Hoechst) were visualised on the fluorescence microscope using a filter with excitation at 365 nm and emission at 420 nm. During each scan, which consisted of a selectable meander pattern from 4 × 4 to 12 × 12 fields, according to the size of the tumour, the previously recorded threshold value was used to isolate the perfused area from the background in a black and white image. After each scan, a composite image of the perfused areas was reconstructed from the recorded and processed black and white images of each field. After immunohistochemical staining for hypoxia and vasculature, sections were scanned on the image analysis system. A filter combination of 450–490 nm excitation and 520 nm emission was used for hypoxia and a filter with excitation at 510–560 nm and emission at 590 nm was used to analyse the vasculature. The sequentially recorded composite images of the tumour sections with detected perfused areas, hypoxia and total tumour vascular bed, respectively, were displayed using pseudocolours for each structure. Lipid droplets were visualised by light microscopy, and the amount of the droplets was qualitatively estimated: low (+), high (++).

A multiparameter analysis of vascularity, perfusion and hypoxia in tumour sections was finally performed. Measured parameters were: the perfused fraction (PF, area of perfused vascular structures divided by the total vascular area), the relative vascular area (RVA, total vascular area divided by the total tumour area), the hypoxic fraction (HF, total hypoxic area divided by the viable tumour area). The number of vascular structures per mm^2^ (VD) as well as the perfused vascular density (VDp) were also calculated.

For each tumour section, the composite images with vascular structures, perfused areas and hypoxic regions were combined, resulting in a new image with the overlapping structures representing the three parameters. In randomly selected regions of interest (approximately 10 ROIs per tumours), each including at least one perfused vessel near a hypoxic region adjoining tissue necrosis, distances from perfused vessels to necrotic areas, crossing hypoxic regions, were measured in manually drawn rectangles. In addition, pimonidazole-binding intensity as a function of distances to perfused vessels was measured using a 12-bit image analysis system (Rijken *et al*, 2000).

## RESULTS

### Occurrence of necrosis and lipid droplets

Table 1

**Table 1:** Tumour characterisation after HE and lipid droplets staining

	**Macroscopic analysis**	**HE**	**Lipid staining**
**Rats**	**Tumour area** (**mm^2^**)	**Necrosis** (***R* %**)	**Lipid droplets**
1	42.8	30.9	++
2	41.2	36.9	++
3	16.9	1.2	+
4	33.2	42.2	++
5	23.5	5.8	+
6	7.3	18.5	++

**Figure 1:**
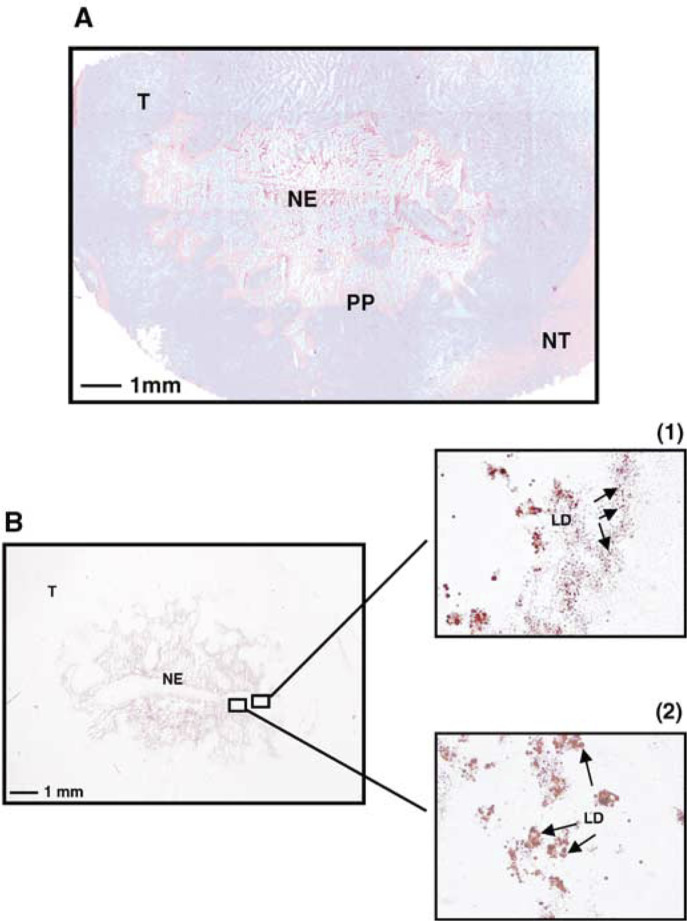
Haematoxylin and eosin (**A**) and Red Oil (**B**) staining of a rat brain section observed 25 days postimplantation. The tumour exhibited an extensive area of necrosis limited by cells arranged into pseudopalisade (A). Lipid droplets were observed mainly in the necrotic zone and also in the cytoplasm of viable tumoral cells surrounding necrosis (B). Enlargements of B show the presence of small lipid droplets (indicated by arrows) in viable cells at the periphery of necrosis (1) and large lipid droplets within the necrosis (2). T=tumour, NE=necrosis, PP=cells arranged into pseudopalisade, NT=nontumoral tissue, LD=lipid droplets.

HE=haematoxylin and eosin staining. *R* represents the necrotic fraction defined as the necrotic area divided by the total tumour area. Lipid droplets: +=low; ++=high.

summarises tumour characteristics after HE and lipid droplets staining. All rats developed tumours characterised by the presence of necrotic tissue. The three largest tumours (rats 1, 2 and 4) exhibited extensive areas of necrosis (respectively, 30.9, 36.9 and 42.7% of total tumour area) surrounded by cells arranged into pseudopalisade (Figure 1A

). A high number of lipid droplets were observed mainly in necrosis, and some smaller droplets were located in ‘pseudopalisadic’ cells (Figure 1B). Two other tumours (rats 3 and 5) showed focal necrotic regions (1.2 and 5.8% of total tumour area) that contained low amounts of lipid droplets. The necrotic area of one of the two rats (rat 5) was surrounded by viable cells arranged into pseudopalisade. Some droplets were visible in this area. The last tumour (rat 6) was characterised by a moderate necrotic area (18.5% of total tumour surface) and a high number of lipid droplets located in the tissue necrosis. Even if no precise quantification was performed, the number and the size of lipid droplets seemed to increase through the pseudopalisade towards the necrotic areas of all tumours.

### Lipid droplets in relation to perfusion, vasculature and hypoxia

**Figure 2:**
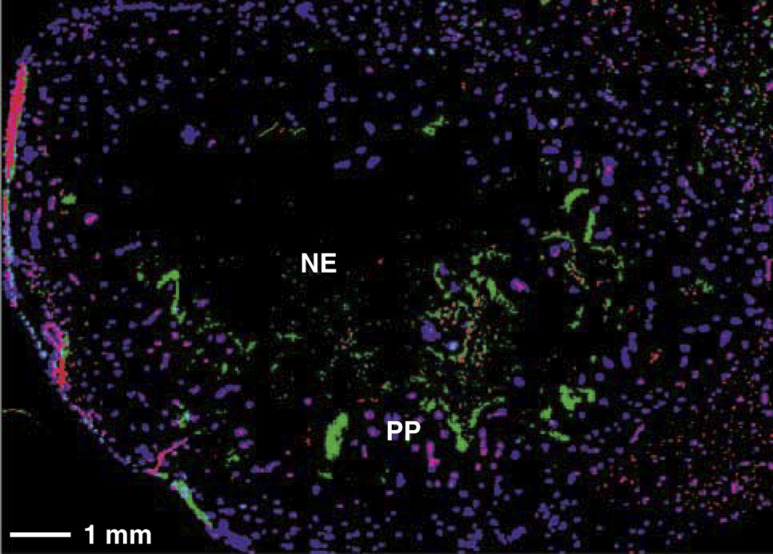
Pseudocoloured composite binary image obtained after sequential scanning for total vascularisation (red), perfusion (blue) and hypoxia (green), respectively, in a rat brain section. NE=necrosis, PP=cells arranged into pseudopalisade.

As is illustrated by the composite digital image in Figure 2

, the vasculature of the tumours was not homogenously distributed. This vasculature (red fluorescence) consisted of a number of large vessels dispersed within the tumour. The necrotic core of the tumour was nearly devoid of vessels. The mean value of the RVA was approximately 0.021±0.019 (Table 2

**Table 2:** Quantification of perfusion, vasculature and hypoxia in rat gliomas

**Rats**	**PF**	**RVA**	**VD**	**VDp**	**HF**
1	0.71	0.011	19.2	14.0	0.029
2	0.28	0.007	13.8	4.2	—
3	0.81	0.024	29.0	20.8	0.023
4	0.60	0.014	20.2	9.3	0.008
5	0.78	0.015	26.5	19.0	0.032
6	0.65	0.055	53.7	30.8	—

Mean±s.d.	0.64±0.19	0.021±0.017	27.0±14.0	16.3±9.4	0.023±0.010

PF=perfused fraction, RVA=relative vascular area, VD=vascular density including all vessels, VDp=vascular density including only perfused vessels, HF=hypoxic fraction. s.d.=standard deviation.

). Hoechst binding (blue fluorescence) was restricted to a small area around the perfused vessels (Figure 2). The multiparameter analysis also showed that more than half of the vessels were perfused: PF represents approximately 64% of the total vascular area (Table 2). In some tumours nonperfused vessels were observed inside necrosis. Hypoxia (green fluorescence) was mainly detected adjacent to necrosis, as shown in Figure 2. However, pimonidazole-binding cells did not uniformly surround the necrosis. In addition, some hypoxic cells were sometimes found within the necrotic tissue. Owing to technical problems, the hypoxic fraction was only measured on four rats out of six. The mean hypoxic fraction was 0.023±0.010 (Table 2). No inverse correlation was found between PF and HF. On the other hand, the density of the perfused vessels seemed to correlate with the hypoxic fraction (Table 2).

**Figure 3:**
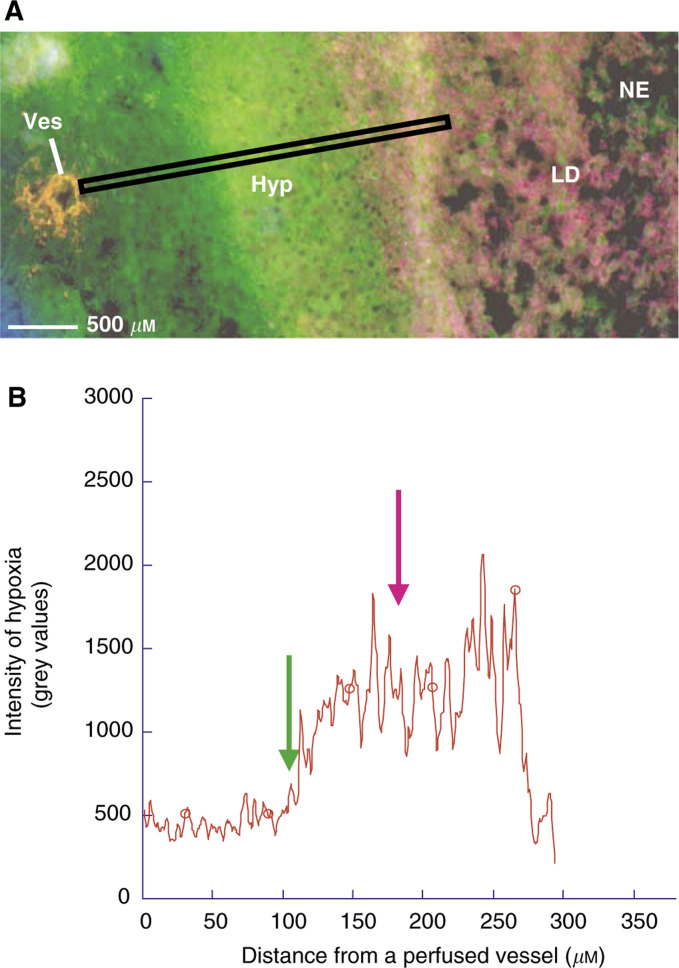
Pseudocoloured 12-bit grey-level image obtained in a selected ROI including at least one perfused vessel near a hypoxic region adjoining tissue necrosis. The black rectangle represents the area in which distances were measured (**A**). The intensity of pimonidazole binding increases from a perfused vessel to the edge of necrosis (**A**). This hypoxic gradient is illustrated in (B); the green arrow shows the mean distance where hypoxia (*p*O_2_<10 mmHg) starts to occur: approximately 114 *μ*m. The pink arrow shows the mean distance where lipid droplets start to occur: approximately 181 *μ*m. Thus, small lipid droplets are present in hypoxic regions, but only in cells adjacent to necrosis (A). Ves=perfused vessel, Hyp=hypoxic region, LD=lipid droplets, NE=necrosis.

Measurements performed on about 40 ROIs selected randomly inside the tumours (Figure 3A

) showed an average value of 114±50 *μ*m for the minimal distance from a perfused vessel to the nearest hypoxic cells. At increasing distance from vessels, pimonidazole binding rapidly increased from background level to a maximum and decreased to background level into the necrotic tissue. An example of this hypoxia gradient is shown in Figure 3B. The maximal intensity of the bound hypoxic marker varied from tumour to tumour and among the different ROIs of a single tumour. For the first rat, for example, the values obtained in 13 ROIs ranged from 402 to 2650 grey values.

Small lipid droplets were observed in hypoxic regions, but only in hypoxic cells adjacent to necrosis (Figure 3A). The average minimal distance between a perfused vessel and the first lipid droplets was approximately 181±72 *μ*m.

Previous studies were performed on 14 other rats (respectively, 4, 5 and 5 rats for each series). The results are not shown here, since the previous immunohistological protocol did not include the staining for functional and permeable vessels. However, similar results compared to those presented here were observed concerning hypoxia binding and the presence of small lipid droplets in the hypoxic cells adjacent to necrosis.

## DISCUSSION

It has been suggested that the presence of lipid droplets in viable cells surrounding necrosis could be related to hypoxia (Freitas *et al*, 1990; Kuesel *et al*, 1994b). The aim of this study was to determine whether these cells were indeed hypoxic and if lipid droplets could be considered as a surrogate marker of hypoxia.

### Localisation of hypoxic regions

Despite the interest in the hypoxic cell detection, methods for reliably and conveniently identifying and quantifying hypoxic viable tumour cells in cancer patients have remained limited (Durand and Raleigh, 1998). Presently, the use of molecules derived from 2-nitroimidazole, which are selectively reduced in the presence of a low oxygen concentration, has been adapted to nonradioactive immunohistochemical assays for the detection of hypoxic cells. Pimonidazole has now been applied to human tumours (Kennedy *et al*, 1997; Varia *et al*, 1998; Raleigh *et al*, 1999). This marker is sensitive to the oxygen variations: it is metabolized at *p*O_2_ lower than 10 mmHg, only in viable cells (Durand and Raleigh, 1998; Evans *et al*, 2000). As no pimonidazole binding occurs in the necrotic areas, it is possible to distinguish these anoxic zones from the hypoxic areas.

Under normal conditions of oxygenation, hypoxia could be related to tumour perfusion and vascularity (Koukourakis *et al*, 2000). Hypoxic conditions can be expected either around a less-perfused blood vessel (acute hypoxia) or beyond O_2_-diffusion distance from well-perfused vessels (chronic hypoxia). In the present study, no inverse correlation was found between hypoxic fraction and perfused fraction (Table 2). This result could be influenced by the presence of areas of necrosis in the tumours. Actually, as illustrated in Figure 2, C6 rat glioma is characterised by a high heterogeneity of vasculature density in the different parts of the same tumour: the necrotic centre of the tumour is completely anoxic and almost devoid of vessels. The intratumour vascular density is higher in peripheral areas than in the central necrotic core of the tumours. Such a vessel distribution was also described in an experimental mouse mammary carcinoma (Thompson *et al*, 1987) and in human carcinomas (lung, breast, colon and endometria) (Koukourakis *et al*, 2000). In addition, the most pronounced vascular density affected the adjacent connective tissue. Consistent with this, our results support the general belief that some tumour periphery in proximity of normal structures are better oxygenated than central tumour areas that suffer from hypoxia and undergo necrosis (Thompson *et al*, 1987). This implies a diffusion-limited hypoxia, determined by the spatial distribution of the perfused vessels. The values obtained for the relative vascular area in each tumour (Table 2) are very low compared to the results described in the literature (0.052–0.2), for human glioma xenografts (Rijken *et al*, 1995, Rijken *et al*, 2000). This result could also be explained by the variation in the angiogenic profile of the different sorts of tumours analysed (Rijken *et al*, 1995; Koukourakis *et al*, 2000).

In C6 rat glioma, chronic hypoxia occurs at a minimal distance of 114 *μ*m from a perfused vessel, a value in the range of those already described in the literature (100–200 *μ*m) (Vaupel *et al*, 1989; Rijken *et al*, 2000). From this distance, pimonidazole binding was found to increase from background level to a maximum at the far end of a hypoxic region (Figure 3A), resulting in a hypoxia gradient. Beyond the critical distance of 114 *μ*m, cells might become more and more hypoxic, some of them might die from the lack of oxygen and nutrients, leading to regions of necrosis completely devoid of oxygen (anoxic regions). This decreasing gradient in oxygen concentration from vessels has also been reported in *in vitro* studies (spheroids) (Franko *et al*, 1992), and *in vivo* studies (tumour tissue) (Franko *et al*, 1987; Kennedy *et al*, 1997; Parliament *et al*, 1997). Although in C6 gliomas elevated pimonidazole binding is mainly found in some areas adjacent to necrosis (Figure 2), it should be noticed that hypoxia does not form a continuous rim around the necrosis. This contrasts with the classical finding of heavily labelled cells uniformly surrounding necrosis in murine tumours (Chapman *et al*, 1983).

In their report, Parliament *et al* (1997) also described unexpected anomalous patterns of nitroimidazole binding adjacent to necrosis in human glioma xenografts. The finding of some regions around the necrotic tissue that are not heavily labelled raises the possibility that necrosis can occur despite an absence of significant hypoxia (Parliament *et al*, 1997). This result could be explained if we considered that the rate of oxygen consumption might be different according to the regions observed. Hypoxic regions around necrosis should represent ‘oxygen conformer cell’, whose rate of oxygen consumption varies with the availability of oxygen (Parliament *et al*, 1997). In some microregions, necrosis may occur despite an absence of notable hypoxia through the diminution of substrates other than oxygen, such as glucose (aerobic and/or anaerobic glycolysis) (Parliament *et al*, 1997). The variation in maximal pimonidazole-binding intensity between tumours and within ROIs is another indication for the existence of ‘oxygen conformer cell’. This variation could also be explained by a variation in capability of tumour cells to bind pimonidazole, if it is considered that this fixation depends on the concentration of fixing protein. Thus, our results show that the degree of hypoxia is not always equal in different tumours observed at the same stage of development, as well as in various zones of a tumour.

### Hypoxia and lipid droplets

Our results revealed the presence of lipid droplets in the cytoplasm of tumoral cells in a few layers of cells surrounding necrosis. Visual evaluation showed an increase in the number of these droplets from the periphery of the necrosis towards the centre of the tumours. The larger droplets were located in the necrotic core of the tumours and the smaller droplets were present in the hypoxic cells surrounding necrosis. However, these droplets were not observed in all hypoxic cells, rather in the most hypoxic cells adjacent to necrosis. This could be explained by the existence of a progressive degeneration of mitochondria as the hypoxic gradient increased. In 1990, Freitas *et al* already showed that lipid droplets were associated with degenerating mitochondria in Ehrlich carcinoma (Freitas, 1990; Freitas *et al*, 1990). At the distance from a perfused vessel where hypoxia (*p*O_2_<10 mmHg) starts to occur (approximately 114 *μ*m according to our results) mitochondrial structures might still allow the activity of the respiratory chain, avoiding the production of lipid droplets. Beyond this critical point, mitochondria might degenerate as the availability of oxygen decreased, leading to a complete disintegration at *p*O_2_ below a certain value corresponding to an approximate distance of 181 *μ*m from a perfused vessel, according to our results. Hypoxic cells might thus produce lipid droplets as a temporary (or end point, if the tissue does not recover) storage compartment for lipids (Freitas *et al*, 1990; Rémy *et al*, 1997). Hence, lipid droplets occurrence seemed to depend on oxygen concentration below a relatively low *p*O_2_ value. This hypothesis is strengthened by recent results of Scarfo *et al* (2001). By studying systemic and pulmonary endothelial cells exposed to acute and/or chronic hypoxia, the authors observed an induction of lipid bodies which was O_2_-concentration dependent. Exposure to 0% O_2_ caused obvious induction of lipid bodies in the cytoplasm of the cells. Exposure to less severe hypoxia (10% or 3% O_2_) also induced formation of lipid bodies observable with Red Oil staining. However, in this latter case, the number of lipid bodies appeared fewer than that observed at 0% O_2_ (Scarfo *et al*, 2001). This could explain the accumulation of lipid droplets in the anoxic necrotic center of C6 tumours, and the presence of a smaller number of droplets inside the hypoxic cells surrounding this necrosis. Therefore, the presence of lipid droplets in cells could be considered as an indicator of a severe, prenecrotic hypoxia, and could be useful for the stratification of tumours in nonhypoxic, hypoxic and severe hypoxic regions to get a targeted treatment for each zone.

The ‘hypoxic’ lipid droplets certainly contribute to the signal of mobile lipids detected by *in vivo* ^1^H MRS. But, these droplets often represent a smaller percentage compared to large droplets localised in the necrotic tissue. Consequently, the occurrence of MRS lipid signal could be considered as an indicator of necrosis and/or severe hypoxia. Now, the problem is to distinguish between the signal arising from ‘necrotic’ lipid droplets and the one that comes from ‘hypoxic’ lipid droplets. If this distinction was feasible, it would constitute a noninvasive and nonradioactive method for detecting the presence of necrosis and revealing the existence of hypoxic zones within a tumour.

## CONCLUSION

The present study shows that at late stages of C6 rat brain glioma development, hypoxia (*p*O_2_<10 mmHg) occurs at a distance of about 100 *μ*m from a perfused vessel, and is mainly detected around the necrotic area. The hypoxia marker binding intensity is considerably variable between tumours and within the different regions of the same tumour. This information on the degree of hypoxia could be useful in tumour treatment. Indeed, a variable dose of irradiation could be applied according to whether the tumour is more or less hypoxic.

Small-sized lipid droplets are not detected in all hypoxic cells but only in the highly hypoxic, prenecrotic ones. As a consequence, lipid droplets could only be associated with a severe, prenecrotic hypoxia and cannot be considered as a surrogate marker of a moderate hypoxia.
